# Correspondence

**Published:** 1859-02

**Authors:** 


### CORRESPONDENCE.

MENDOTA, ILL., Feb. 24, 1859.

Dr. N. S. DAVIS—

DEAR SIR: I have had a case, for more than two years, which, I apprehend, is of more than ordinary importance; and to make a correct diagnosis of which, to me, is a matter of great difficulty. I will, as briefly as possible, give you a sketch of the case, and shall thankfully receive any light which you may kindly give me in regard to it.

*Feb. 21st*, 1857.—Was called to see Mrs. G., aet. 30. Supposed herself to be in her third pregnancy. Had ceased to menstruate Oct. 27, 1856. Had had symptoms similar to those of her previous pregnancies—cessation of courses, morning sickness, swelling, soreness and secretion in breasts. The day previously to my being called, had exerted herself rather more than usual; this morning had pretty severe periodical pains in lower part of bowels, with some hemorrhage; this becoming worse, I was called. From a history of her case and present condition, was at once led to think that she was pregnant, and threatened with a miscarriage. Resorted to the ordinary treatment in such cases, and in the course of 48 hours the flowing had entirely ceased, but still some pain, which continued more or less for four or five days. As she is a woman of a rather delicate organization and highly excitable nervous temperament, rest was advised, and the horizontal posture as much as possible. At the expiration of the four months, she thought she felt motion, or the quickening, and declared that she felt more or less movement, which was also affirmed by her husband; the movement being quite distinct, though not so strong as had been in her previous pregnancies. Up to July 26th, which would make the nine months, the abdomen gradually and uniformly enlarged, and at the expiration of that time was about the usual size of a woman of her developments at full term.

*July 26th.*—In my absence, another physician was called to see her, as she supposed herself to be in labor. Had considerable show, and frequent, severe pains. He was soon convinced that she was not in actual labor, and administered an anodyne; the pains soon subsiding.

She ran along then, without changing in size, feeling no movement, but suffering from pains, which she compared to the pains of the first stage of labor, occasionally, but most severely at the expiration of every month. Her general health improved, and she was able to take charge of her domestic affairs.

In Sept., 1857, the catamenia returned, and she has been perfectly regular ever since. The case did not then receive any especial attention from me until Jan. 31, 1858. Mr. and Mrs. G. becoming still more uneasy as to her condition, I was requested to call, and, if possible, to give a positive opinion as to the nature of the case. I would here remark, that up to the expiration of the nine months, I had scarcely any suspicion whatever but that the case was one of ordinary pregnancy; and as the case still did not terminate up to the 1st of October, I thought that possibly there was some miscalculation. After that time, the case became one of peculiar interest, the conviction becoming stronger and stronger that she could not be pregnant. Now of her condition Jan. 31, 1858:

Looked very well; countenance cheerful; in usual flesh; appetite very good, bowels regular, menstruates every month. Does not suffer as much at those times as she formerly did; occasionally has some pain in the lower part of her bowels—more at nights. The enlargement of abdomen uniform; no change in size. Nothing revealed by stethoscopic examination. Could get the outlines of the uterus distinctly; fundus rather more to left side than right; no unusual sensitiveness on either pressure or palpation; quite firm; change of position of body does not change position of tumor. Dull sound on percussion, no fluctuation.

*Examination per vaginum.*—Could very easily reach the body of the womb anteriorly; rested very low in the pelvis. Os uteri central in vagina, with probably one inch of cervix projecting into vagina; quite smooth, but very firm; the body and all of the cervix, excepting as much as referred to, uniformly developed.

Os uteri very small; could not introduce tip of index finger. Could not, by pressure, and with one hand applied to the abdomen, elevate the uterus.

*Feb. 16th,* 1859.—No perceptible change in her condition in any respect, since Jan. 31, 1858. Would see her occasionally, and learn that she "was quite smart"—as well as when in her best health. For the last two weeks has been suffering considerably with pain over the cotyloid cavity, and for several nights extremely with pains resembling first stage labor pains. Re-examined the case carefully; no change in size since last examination. Digital examination—os easily reached; directed rather backward, small and contracted; the tumor firmly fixed in the pelvis. Digital examination per rectum, and vaginal examination with speculum—nothing revealed worth noting. The difficulty must be intra-uterine, or involving the whole uterine parieties. To take anodynes pro re nata.

*Feb.* 23.—During the last week, when suffering severe contractile pains of the uterus, there has been, at several times, a slight escape of fluid from the os, probably a half ounce at a time.

From all the information I have been able to gain, by consulting authors and physicians, I must regard this as rather an unique case. I have briefly but accurately stated all the important particulars of the case. The sequela alone will determine what the *trouble* is. But I assure you, that if you, or any to whom you may refer, can give me any light in regard to the case, it will be thankfully received. Am I right in pursuing the let-alone course? Or would I be justified in introducing an exploratory instrument through the os, and, if possible, puncture the uterine contents, if such there be. I shall be glad to hear from you at your earliest opportunity. The case will be worth reporting, when it terminates, and if you should not consider it premature, the preceding notes are at your disposal for the *Journal*. I am yours truly,

E. P. COOK.

---

HAMPDEN, *Rock Island Co., Ill.*

PROF. N. S. DAVIS—

DEAR SIR: In the August number of your Journal, you request the readers thereof residing in the country districts, to favor you with an account of the special characteristics of fevers prevalent in those districts.

Throughout this section of country, fevers, as presented to my observation and attention thus far, this fall, have assumed a more aggravated type than has ever before been known. A striking characteristic seems to be the apparent tenacity in the diseases, not yielding so readily to remedial measures as in former seasons. Other features are presented in the complication with organic diseases of various organs—the typhus and typhoid conditions supervening upon the mildest remittents and intermittents; the unusual prevalence of fevers of a quotidian type, presenting what is commonly termed congestive, but more properly pernicious, symptoms; hepatic derangements to a great extent; dysentery; diarrhœa; profuse and unnatural diaphoresis; greatp rostration after the febrile spasm was spent—constituted the principal peculiarities of fevers prevalent during August and September.

In two cases in which the tuberculous diathesis was present, the vital activity was so much lowered, that death ensued in a few hours, in consequence of the complete disintegration of the lung tissue, rapidly filling up the air passages, and causing strangulation, the patients being unable to expectorate the disorganized matter as fast as it formed. Another case, presenting the same diathesis, was taken down with fever of a remittent type, followed by great prostration, whom I kept supported by alcoholic stimulants for two or three weeks. The patient recovered without presenting any symptoms of advancement of the tuberculous disease, not having even so much as a cough. Query—would not the same treatment have saved the lives of the other two? I am somewhat inclined to think that it would. I merely state these facts without entering into any speculations in regard to the use of alcoholic stimulants in phthisis. Much has been said and written upon the subject, but I am inclined to believe that much less has been done in the way of trial. I would like to hear the experience of your many readers upon the subject.

J. R. HAYES.

---

### NOSTRUM VENDERS.

Hope Dale, Illinois.

Eds. Chicago Medical Journal:

It is to me a matter of much regret, that in the popular and wealthy county of Tazewell, represented as it is by so many intelligent members of our profession, there is not, nor to my knowledge has there been, an association for the mutual improvement, regulation, and intercourse of the members of the medical profession: Nor does it seem an easy matter to bring physicians, who have been practicing for many years, to an appreciation of the great importance of, and the many benefits arising from, local organization, *here.* In extenuation of myself, I would say, that I have urged this matter, time and again, to the consideration of the profession in my vicinity, but without avail. In short, I have done everything consistent for one of my age and influence, to bring about this desired object. Now, what is the reason of this apathy on our part? What is it that so like a narcotic drug allays our sensibilities, and invites to rest every attention to our interests but those of *lucre*, to every care for mental and professional advancement, and to the proper elevation and maintenance of the importance and dignity of our most noble profession? What is it that causes our profession, in this community, to maintain so long a profound silence on this important and vital subject? What is it that keeps its members in the old routine, maintaining the principle of "let every man take care of himself?" Can it be indolence and lack of care for the interests of the profession? The true physician is ever active and awake to the duties he owes to himself and his profession. Can it be that many of those to whom we would look as pioneers in this enterprise, fear the exposition of the extent of their qualifications, which associations would likely induce? I hope not. Can it be that there are too few readers of the *Chicago Medical Journal* in our county? Can it be that jealousy, caused by opposition to each other in practice, or the attention paid to the recent play of Bowie knives on the ribs of each other (of two of the profession amongst us) has usurped the place of interest in other and more honorable things? Or can it be, Messrs. Editors, that many of those to whom, from their age and experience and intelligence, we are expected to look as proper lights in our profession in this county, are to-day, and for some time past have been, engaged in the reprehensible and dirty work of selling and dispensing *patent nostrums?* Please send the readers of the *Journal* your opinion of these things—of this one in particular: Whether physicians, with diplomas from respectable medical colleges, who vend and sell secret or patent medicines, on commissions or otherwise, should be considered as holding an honorable position in a profession one of whose tenets is to discard all connection with illegitimate medicine, and especially all sympathy with venders of *quack nostrums* and patent remedies?

Please accept the within remittance as your dues for the present volume of the *Journal;* and if the above imperfect, and mayhap rather harsh, exposition of some things that do exist in this community, would be read and found to fit the case of any person or persons claiming to be physicians, and who are living on the proceeds of the sale of quack nostrums; and if in your judgment it deserves a place in your columns, please publish the same. P. W. HILL, M.D.

---

APPOINTMENTS IN MEDICAL SCHOOLS.

Dr. A. G. Thomas has been appointed Professor of Physiology in the Oglethorpe Medical College, Georgia.

Dr. John C. Draper has been appointed to the Professorship of Analytical and Practical Chemistry in the University of New York.—*N. American Medico-Chirurgical Review.*

